# Intra- and inter-rater reliability of thoracic spine mobility and posture assessments in subjects with thoracic spine pain

**DOI:** 10.1186/s12891-020-03551-4

**Published:** 2020-08-10

**Authors:** Jani Takatalo, Jari Ylinen, Tuomo Pienimäki, Arja Häkkinen

**Affiliations:** 1 Faculty of Sport and Health Sciences, University of Jyvaskyla, Jyväskylä, Finland; 2 Medical Research Center Oulu, University of Oulu and Oulu University Hospital, Oulu, Finland; 3 Central Finland Central Hospital, Jyväskylä, Finland; 4 The Social Insurance Institution of Finland, Helsinki, Finland

**Keywords:** Thoracic, Spine, Pain, Reliability, Clinical examination, Manual therapy, Observation, Inclinometer, Tape measure, Palpation

## Abstract

**Background:**

The thoracic spine (TS) has been neglected in the study of the spine despite its essential role in the stability and posture of the entire spinal complex. Therefore, there is an inevitable need to investigate the reproducibility of different thoracic spinal posture measures used in subjects with TS pain.

**Methods:**

Thirty-two subjects (16 females and 16 males, mean age 39 years) were evaluated by two physiotherapists on the same day to gauge inter-rater reliability and on two consecutive days to gauge intra-rater reliability. TS posture was assessed by observation, and thoracic spine mobility was measured by manual assessment of segmental flexion and extension mobility in a seated position. Additionally, posterior-to-anterior accessory mobility in a prone position was assessed manually. Moreover, cervicothoracic flexion in a seated position, thoracic posture, and thoracic flexion and extension mobility in a standing position were assessed with a tape measure, and flexion and extension mobility in a seated position and TS posture in seated and standing positions were measured with an inclinometer. The intraclass correlation coefficient (ICC), standard error of measurement (SEM), mean difference (MD), Bland-Altman (B&A) plot features and coefficient of repeatability (CR) were calculated.

**Results:**

The mean and standard deviation (SD) of the duration of TS pain was 22 (SD 45) months, with the intensity of pain being rated at 27 (SD 21) mm on a visual analogue scale (VAS). Intra-rater reliability was very strong (ICC ≥ 0.80) for the evaluation of seated and standing upper TS posture, standing whole TS posture and seated lower TS posture with an inclinometer. Moreover, TS posture evaluation with a measuring tape, posture inspection in a seated position, and manual assessment of segmental extension were found to have very strong intra-rater reliability. Inter-rater reliability was very strong for inclinometer measurements of standing and seated upper TS posture as well as standing whole TS posture.

**Conclusion:**

Intra-rater reliability was higher than inter-rater reliability in most of the evaluated measurements. Overall, posture measurements with an inclinometer were more reliable than mobility measurements with the same instrument. The manual assessments can be used reliably when same evaluator performs the examination.

**Trial registration:**

Clinical Trials, NCT01884818. Registered 24 June 2013, https://clinicaltrials.gov/ct2/show/NCT01884818?cond=thoracic+spine&cntry=FI&rank=1

## Introduction

One-fifth of all people suffer from thoracic spine pain in their lifetime [1]. Niemeläinen et al. [2] found in their survey that 17% of males reported TS pain, with half of that subset reporting severe thoracic spine pain (visual analogue scale [VAS] rating > 80 from 100 points on a numerical pain scale). One-quarter of these males experiencing thoracic spine pain had difficulties in daily activities. Female sex and younger age (children and adolescents) are also risk factors for thoracic spine pain. However, the prevalence is highly variable, ranging from 4 to 72% across different studies [1–3]. Patients with thoracic spine pain often undergo clinical evaluation to assess the need for therapy and measure its results [4]. Thus, it is necessary to study the reliability of the clinical methods for thoracic spine evaluation.

The thoracic spine is the stiffest part of the vertebral column due to the structural differences compared to the cervical and lumbar spine but also due to the thorax [4–7]. The thoracic spine contributes to cervical spine movements [8], flexion of the glenohumeral joint [9] and movement of the ribcage in respiration [10]. All innervated structures in the thoracic spine are capable of providing nociception. If active movements provoke pain, the movements are usually thoracic spine rotation or/and extension [11]. Moreover, shoulder, upper limb and neck pain, and headaches, which are more commonly considered to originate from the cervical spine, may be referred from the thoracic spine instead [12–14]. In the half of all cases of thoracic spine pain seem to involve facet joints [15]. Unilateral thoracic spine pain from symptomatic thoracic spine facets is typically referred one or two segments cranially or caudally [16]. However, the two highest and three lowest thoracic facet joints often refer pain more widely and atypically than the others [11, 17].

Manual examination of the spine has been questioned as the target tissue of examination is controversial. Moreover, only a small number of studies have been published on manual palpation testing of the thoracic spine. In a recent study, Beynon et al [18] found interrater reliability of thoracic spine stiffness ranging between − 0.11 and 0.53 with Kappa statistics. In the review on spinal motion palpation, only a few thoracic spine studies were referred and they found the intra-rater agreement of segmental thoracic spine flexion in sitting and posterior-to-anterior (PA) pressure assessment to be higher than 93%,while in another study PA pressure assessment kappa values ranged from 0.43 to 0.55 and from 0.14 to 0.35 in intra-rater and inter-rater evaluation, respectively [19]. In more recent review on spinal motion palpation concluded, that there are no one superior manual method for physical assessment. Only one study on thoracic spine palpation was included which found kappa values of 0.34–0.77 and 0.38–0.70 for intra-rater and inter-rater reliability, respectively [20]. Overall, it is more reliable to find the same painful thoracic spine segment than segment with movement dysfunction or restriction [18, 20]. Although the movement dysfunction may be found, naming it similarly between raters or between evaluation sessions by the same rater is more challenging [19].

Several measurement devices have been used to measure the posture and mobility of the thoracic spine in the earlier studies. In the systematic review of thoracic spine posture found that most of the studies have been performed with asymptomatic subjects [21]. They also found that reliability may be excellent (Intra-Class Coefficiency, ICC, or Cronbach’s alpha > 0.80), however, validity has been less studied across the large variety of measurement devices. Tape measure has been used mainly in Schober measurement in the lumbar spine. In the lumbar spine flexion and extension intra-rater reliability (ICC) has been reported to be higher than 0.72 [22].

In clinical framework, examining spinal range of motion has been an important part of manual therapy practice [23, 24]. It has been reported that 98% of manual therapists evaluates passive movement of spinal segments as part of their clinical assessment [24]. The major deficiency of earlier studies is a small sample size or without sample size calculations, wide variations of palpation protocols and measurement devices, and mostly performed with asymptomatic subjects.

The thoracic spine, although neglected in the study of the vertebral column, is nonetheless an important part of the spine and can cause severe disability and pain if injured. Therefore, the reliable methods for clinical examination of thoracic spine are important. The aim of the current study is to investigate the intra- and inter-rater reliability of thoracic mobility and posture measurements taken manually and with measuring devices on subjects with cervicothoracic symptoms in order to increase knowledge of reliable measurements with which to investigate thoracic spine problems.

## Methods

The subjects were referred for physical therapy by a physician due to thoracic spine pain or were recruited through advertisement in the local newspaper in the city of Oulu. The physician screened all the potential subjects for other medical conditions before their enrolment. The physical therapist of an outpatient clinic screened the volunteer participants for eligibility before the first visit. Each participant signed informed consent before the first assessment. The inclusion and exclusion criteria are listed in Table 1. The present study was nested cohort study of the randomized controlled trial. The study was conducted according to the Declaration of Helsinki, and the Ethical Committee of Northern Ostrobothnia approved the protocol prior to the study.

**Table 1:** Inclusion and exclusion criteria of the study

Inclusion	Pain in thoracic spine area during baseline examination (VAS > 0)
	Daily thoracic spine pain during the last week(s)
	Pain produced in PA pressure test of the thoracic spine
	18 to 55 years old^a^
Exclusion	Fibromyalgia
	Daily cervical or lumbar spine pain
	Inflammatory musculoskeletal diseases (i.e. rheumatoid arthritis, polymyalgia rheumatic) or any infection
	Spinal fracture or malignant disease
	Haemophilia or other blood disease
	Symptomatic angina pectoris
	Operation with thoracotomy
	Previous thoracic spine operation
	Cardiac pain
	Oesophageal pain

*VAS* Visual Analogue Scale; *PA* Posterior to anterior

^a^ This study was part of the larger thoracic spine manipulation study and therefore upper cut-off age was 55 years of age

**Table 2:** Thoracic Spine Pain and Disability questionnaire

Question	Pain and disability scale from 0 to 100 mm
How severe is your pain?	No pain – intolerable
How severe is your pain at night?	No pain – intolerable
Do you get relief from painkillers?	Complete relief – no relief
How stiff is your thoracic spine?	No stiffness – intolerable stiffness
Do you have discomfort when looking upwards?	None at all – intolerable
Do you have discomfort when turning your head to the sides?	None at all – intolerable
Does your pain interfere with your ability to work with your hands overhead?	No interference – completely unable to work with hands overhead
Does your pain interfere with your ability to comb your hair?	No interference – completely unable to comb hair
Does you pain interfere with your ability to put on your coat?	No interference – completely unable to put on coat
How severe is your pain when lying down in bed?	No pain – intolerable
What is your overall handicap in your complete lifestyle because of your pain?	Completely free to perform any task – totally handicapped
To what extent does your pain interfere with your work?	No interference at all – totally incapable to work
To what extent have you had to modify your work in order to be able to do your job?	No adjustment to work – so much adjustment that you have had to change your job

Pain and disability was indicated during the last week. The values of each question were summed up, and the total score of the questionnaire ranged between 0 and 1300 mm

The Thoracic Spine Pain and Disability questionnaire is modified from the Neck and Shoulder Pain and Disability questionnaire (Viikari-Juntura et al. 1988)

The enrolled subjects completed a 24-h thoracic spine pain questionnaire (VAS, from 0 = no pain to 100 = worst **pain** imaginable), the Roland-Morris Disability Questionnaire (R-MDQ; 0–24 points) and the Thoracic Spine Pain and Disability (TSPD) questionnaire (sum of 13 items on a scale of 0–100 mm) before each measurement day (Table 2). The questions on the TSPD were modified from the Neck and Shoulder Pain and Disability Index questionnaire [25] to apply to the thoracic spine region. The pain and disability questionnaires were used to obtain a general overview of the severity of the subjects’ thoracic spine pain and their level of disability. Subjects were informed that they would receive feedback about the assessments after the final measurement. Moreover, in order to promote compliance among the subjects, they were rewarded with a 30-min massage to be redeemed after the study.

### Measurement procedure

Examinations were performed on two consecutive days by two physical therapists, who had been trained to perform all the measurements according to the same protocol. The physical therapists were blind to each other’s results and to the results of the pain and disability questionnaires. On the first day, a physical therapist (JT) with 7 years of experience in manual therapy screened the subjects for eligibility and performed the first examination. On the following day, two similar sets of measurements were performed (by JT and JM) in a random order, with ten-minute intervals between assessments. The physical therapist performing the measurements on consecutive days (JT) was blind to the recordings of the initial measurement on the second examination. Each subject was examined at approximately the same time of day and the order of the examinations were similar on consecutive days. The other assessing physical therapist (JM) had 23 years of experience in manual physical therapy. Each mobility examination was performed maximum of two repetitions for each segment to reduce the mobilizing effect of assessment.

### Preparations for the assessment

Before each assessment, the subjects were asked to sit, while the physical therapist palpated and marked the spinous process of the C7 vertebra with a pen. The spinous process of C7 was identified by cervical extension as spinous process of C6 appear to move anteriorly and C7 remains stationary during the movement [26]. Without extension C6 is found to be the most prominent spinous process in 48% of the subjects, but with extension test the spinous process of C7 can be found in 77–88% of subjects [27]. After the C7 spinous process was found and marked, the next mark caudally was drawn on the spine 15 cm from the marking of the spinous process of C7, theoretically representing the spinous process of T5 [28]. The T6 and T12 spinous processes were palpated by palpating caudally the spine and marked with the pen. All marks made on the spine were erased at the end of each assessment session to blind the physical therapists for their previous as well as each other’s markings.

### Assessment of the thoracic spine posture

**Fig. 1:**
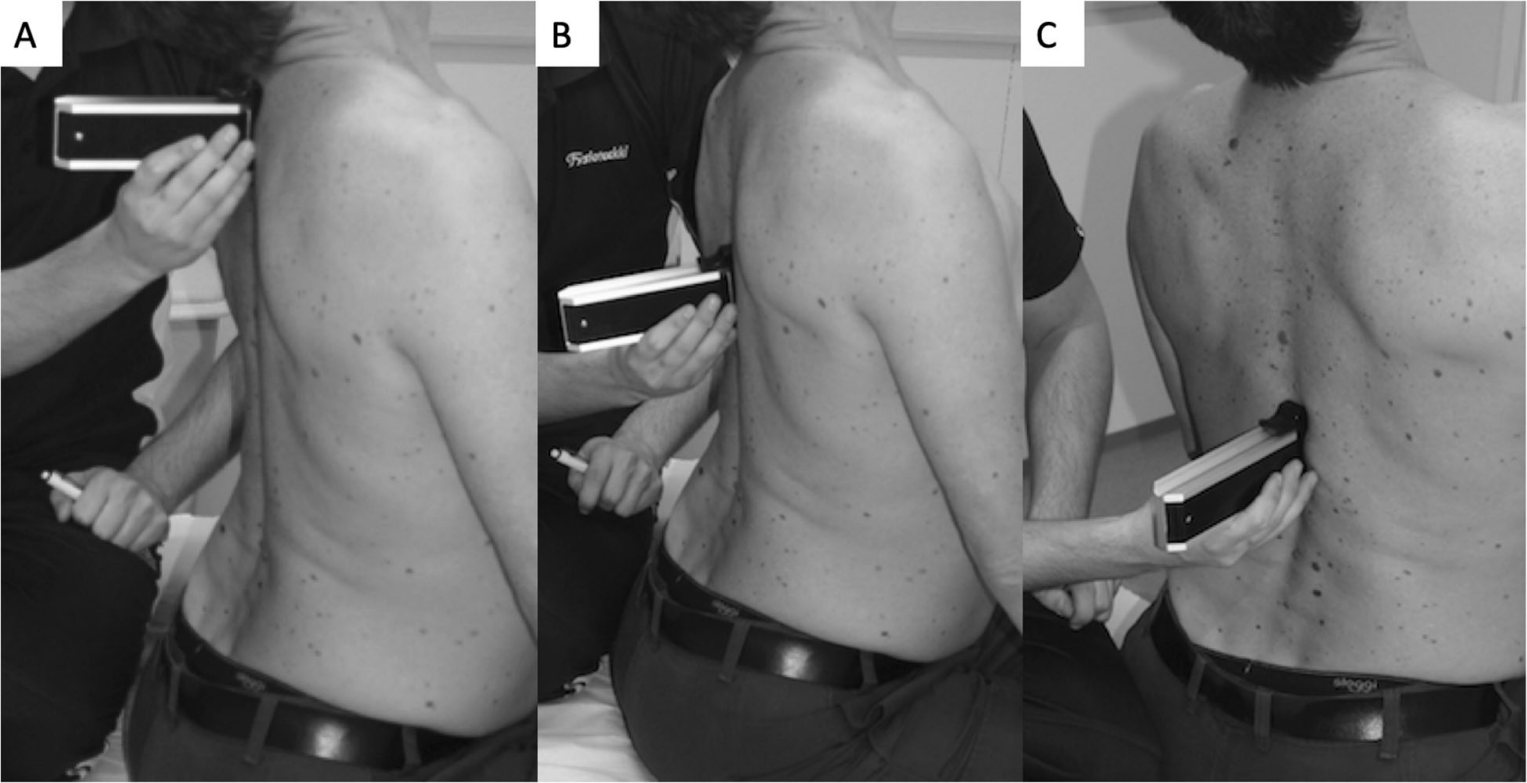
The assessments of the thoracic spine extension with inclinometer in sitting. The assessments were performed in upper thoracic spine (**a**; Th1), mid-thoracic spine (**b**; Th6), and lower thoracic spine (**c**; Th12). Similar measurements were performed in maximum flexion of the thoracic spine. The thoracic spine posture was evaluated in same three locations in sitting and standing while subject maintained his/her typical posture

The subjects were standing with the feet slightly apart (at the subject’s typical standing width); the physical therapists evaluated the subject’s thoracic spine posture from the side view and classified it as hypokyphosis, normal kyphosis or hyperkyphosis. The evaluation was a forced call methodology based on clinical experience of the posture of the thoracic spines, meaning the physical therapist had to classify the subjects’ thoracic spine one of the categories before inclinometer measurements. The inclinometer (Saunders Digital Inclinometer, New York) was used to measure thoracic spine posture at the T1, T6 and T12 levels; for the first two measurements (T1 and T6), inclinometer was placed caudally so that the edge of the upper contact pillar of the inclinometer was in contact with markings, whereas in the third measurement (T12), the edge of the lower contact pillar of the inclinometer was cranial to the T12 marking. The same measurements were performed in a sitting position (Fig. 1); the subject was encouraged to sit in his or her natural position. The actual inclination of the thoracic spine was calculated based on these values for the upper (from T1 to T6), lower (from T6 to T12) and whole TS (from T1 to T12). These measurements of thoracic spine posture have been earlier described by Czapowski et al [29] in standing.

### Assessment of the thoracic spine mobility with inclinometer and tape measure

Active thoracic spine flexion and extension were measured while the subject was sitting. The subject was asked to flex the spine as far as they could despite a possible increase in thoracic spine pain. The thoracic spine flexion was measured with both a tape measure (1) and an inclinometer (2). The upper thoracic spine (C7-T5) mobility was measured as suggested by Norlander et al. [28]; the difference between the marks on spinous processes of C7 and theoretical T5 (15 cm from the marking of C7) in neutral and maximum flexion was recorded. The thoracic spine Schober test was performed in a sitting position from the spinous process of T1 to that of T12. Measurements were performed in a neutral position and in active end-range flexion of the thoracic spine, and the difference between these measurements was recorded as the thoracic spine Schober flexion value. The extension of the thoracic spine was measured similarly by calculating the difference between neutral and active end-range thoracic spine extension tape measurements, and the value was recorded as the thoracic spine Schober extension value [26]. The inclination of T1, T6 and T12 was measured in active end-range thoracic spine flexion and extension to determine the upper, lower and total thoracic flexion and extension. The contact pillars placements of the inclinometer were similar as for thoracic spine posture measurements. The differences between neutral and maximum flexion angles and between neutral and maximum extension angles were used as flexion and extension mobility values, respectively (Fig. 1). As we aimed to measure the maximum available active range of motion in the thoracic spine, we did not prohibit the lumbar movement as it is impossible to do maximum thoracic movement without moving the lumbar spine. Moreover, we were interested in whether the reliability would change if thoracic spine is measured in two halves, as thoracic spine is the longest part of the vertebral column and biomechanics of the upper thoracic spine is different compared to the lower part.

### Manual assessment of the thoracic spine mobility

**Fig. 2:**
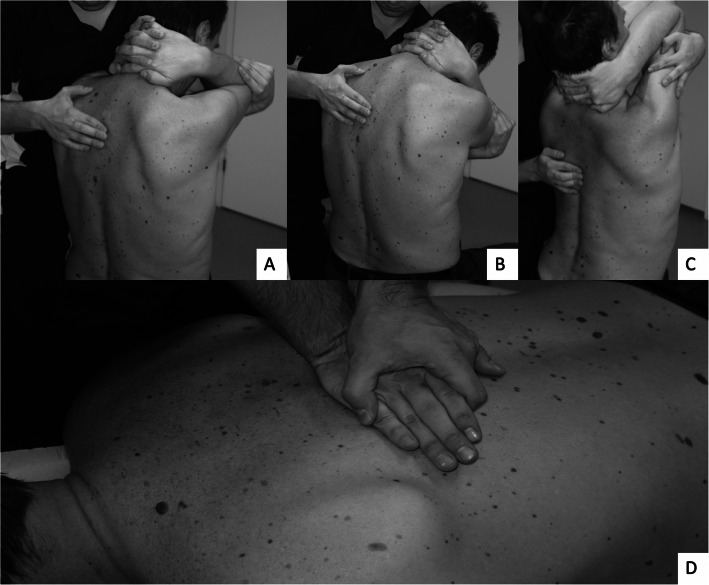
Manual assessment of the thoracic spine mobility. Each thoracic spine movement segment of the thoracic spine was palpated in sitting (**a**-**c**) and prone (**d**). In sitting, segmental evaluation started from the neutral position of the segment (**a**), followed by guided flexion (**b**) and extension (**c**) movement while physical therapist palpated the interspinous space to evaluate the mobility of the thoracic spine segment. In prone (**d**), posterior-anterior pressure was applied to feel the accessory movement of each thoracic spine segment

The segmental intervertebral motion was assessed in the sitting position as the subject was (semi) passively moved from a neutral posture into (1) flexion and (2) extension (Fig. 2) [26, 30]. Subjects were allowed to take part of the movement although the movement was mainly performed by physical therapist. The subject clasped both hands behind the neck and kept the elbows together in front. The physical therapist flexed and extended the thoracic spine through the subject’s elbows with one hand while using the other to palpate the movement between spinal processes. The possible hypermobile segment was recorded as normal. The evaluation was a forced call methodology based on clinical experience of the manual assessment of the thoracic spine and comparing the adjacent movement segment to each other, meaning the physical therapist had to classify each segment whether normal of hypomobile i.e. stiff and evaluation was made in both directions (flexion and extension). The quality of motion of the thoracic spine segments at end-range, i.e. end-feel, was evaluated by applying posterior-anterior (PA) pressure to each spinous process (3) while the subject was prone on treatment table [26, 30, 31], and the results were recorded as either mobile or hypomobile (Fig. 2). As in manual assessment of the thoracic spine flexion and extension, the evaluation was a forced call methodology based on clinical experience of the end-feel of the thoracic spine, meaning the physical therapist had to classify the subjects’ thoracic spine either normal (normal “springiness”) or stiff (no “springiness” or “hard end-feel”).

### Statistics

The number of subjects needed for the current study was based on the two evaluators, with statistical significance being defined as *p* < 0.05, power being 0.20, assuming that the intraclass correlation coefficient (ICC) was at least 0.50 with assumption that the reliability would be 0.80. These assumptions showed the appropriate number of subjects to be at least 22 [32]. The differences in demographic variables between genders were analysed by independent Student’s t-test and Fisher’s exact test. Moreover, paired-samples Student’s t-test was used compare the inclinometer values of thoracic spine posture between sitting and standing. The intra-rater and inter-rater reliability of the thoracic spine posture and mobility assessments was analysed using the ICCs (two-way mixed, average measures, consistency). Intraclass correlation was classified as very weak (0.01 to 0.19), weak (0.20–0.39), moderate (0.40–0.59), strong (0.60–0.79) or very strong (≥0.80) [33]. Moreover, the 95% confidence intervals of the ICC were calculated, along with Cronbach’s alpha. In order to evaluate the amplitude of differences, the standard error of measurement (SEM), the mean of differences and the coefficient of repeatability were calculated. The calculation of SEM was based on standard deviation (SD) and ICC (r); SD $\sqrt{\Big(1}-r\Big)$. Coefficient of repeatability (CR) was calculated by multiplying the SEM with 2.77 [34]. Bland-Altman plots were used to evaluate the 95% limits of agreement among measurements, and linear regression was performed to test the proportional bias of the Bland-Altman plots. The data analyses were performed using SPSS software for Mac (version 24.0, SPSS Inc., Chicago, IL), however, SEM and CR were calculated manually.

## Results

The mean age of the participants (*N* = 32) was 39 (standard deviation, SD 9.4) years, the duration of thoracic spine pain was 22 (SD 45) months, and the mean intensity of pain in previous 24 h (VAS) was 27 mm (SD 21). The subjects’ mean TSPD score was 303 mm (SD 211) The mean R-MDQ score was 2.3 (SD 2.2) out of a maximum of 23 points. The mean body mass and height of the subjects were 73 kg (SD 15) and 167 cm (SD 29), respectively. Males had higher BMI and weighted more than females, but otherwise characteristically genders did not differ from each other. Additional details on the symptom characteristics and demographic data are shown in Table 3.

**Table 3:** The mean and standard deviation (SD) or prevalence of the demographic variables of the study subjects

	Females	Males	Gender difference	All
	*N* = 16	*N* = 16	*p*-value (df)	*N* = 32
Age, years (SD)	39 (10.6)	39 (8.4)	0.956 (30)^*^	39 (9.4)
Height, cm (SD)	164 (5.0)	169 (41.9)	0.637 (30)^*^	167 (29.4)
Weight, Kg (SD)	63.2 (9.6)	82.8 (12.4)	< 0.001 (29)^*^	72.7 (14.7)
Body mass index, Kg/m^2^ (SD)	23.4 (3.3)	25.6 (2.8)	0.053 (29)^*^	24.5 (3.2)
TS pain, months (SD)	24.1 (52.4)	20.6 (37.9)	0.827 (30)^*^	22.4 (45.0)
VAS, mm (SD)	29.1 (22.9)	24.7 (19.2)	0.558 (30)^*^	26.9 (20.9)
R-MDQ, points (SD)	2.1 (1.8)	2.4 (2.5)	0.689 (30)^*^	2.3 (2.2)
NSPD, points (SD)	316 (211)	291 (217)	0.738 (30)^*^	303 (211)
Smoking, % (N)	12.5 (2)	12.5 (2)	1.000 (1)^§^	12.5 (4)
Physician consultation, % (N)	43.8 (7)	56.3 (9)	0.724 (1)^§^	50.0 (16)
Pain medication for TSP, % (N)	18.8 (3)	6.3 (1)	0.600 (1)^§^	12.5 (4)
Earlier MT intervention, % (N)	87.5 (14)	93.8 (15)	1.000 (1)^§^	90.6 (29)
Systemic disease^#^ % (N)	12.5 (2)	25.0 (4)	0.654 (1)^§^	18.8 (6)

*df* Degree of freedom; *TS* Thoracic spine; *VAS* Visual analogue scale; *R-MDQ* Roland-Morris Disability Questionnaire (maximum points 24); *NSPD* Modified Neck and Shoulder Pain and Disability questionnaire (maximum score 1300, thirteen questions about pain and disability, each evaluated on VAS from 0 to 100 mm); *MT* Manual therapy

^*^ Independent T-test was used for gender differences

^§^ Fisher’s exact test was used for gender differences

^#^ At least one reported systemic disease

### Thoracic kyphosis angulation differences in sitting vs. standing

In inclinometer-based posture measurement, there was no significant difference between the standing and seated posture of the upper thoracic spine, but lower and whole thoracic spine kyphosis were straighter in a seated position than in a standing position (9° vs. 11°, *p* = 0.008, for the lower thoracic spine and 28° vs. 32°, *p* = 0.001, for the whole thoracic spine, respectively). In the lower thoracic spine, a small but significant difference (7.7° vs. 9.4°, *p* = 0.022) was found in females but not in males. In the whole thoracic spine, a significant postural difference was found in males (31° vs. 36°, *p* = 0.006) but not in females. Further details are shown in Table 4.

**Table 4:** The mean, SD and range of the thoracic spine posture and mobility measurements

	Females *N* = 16		Males *N* = 16		All *N* = 32	
	Mean (SD)	Range	Mean (SD)	Range	Mean (SD)	Range
Thoracic posture, standing (°)^a^						
Upper (Th1–6)	17.0 (5.4) *p* = 0.774, df 15	5–25	22.9 (4.0) *p* = 0.193, df 15	16–31	19.9 (5.6) *p* = 0.329, df 31	5–31
Lower (Th6–12)	9.4 (4.6) *p* = 0.022, df 15	0–16	13.2 (4.2) *p* = 0.062, df 15	6–20	11.3 (4.8) *p* = 0.008, df 31	0–20
Upper and lower (Th1–12)	27.0 (7.2) *p* = 0.114, df 15	12–40	36.1 (4.5) *p* = 0.006, df 15	26–44	31.5 (7.5) *p* = 0.001, df 31	12–44
Thoracic posture, sitting (°)^a^						
Upper (Th1–6)	17.3 (5.0)	5–23	21.0 (8.1)	5–38	19.1 (6.9)	5–38
Lower (Th6–12)^b^	7.7 (4.9)	−3 – 15	9.9 (7.3)	−3 – 22	8.8 (6.2)	-3 – 22
Upper and lower (Th1–12)	24.9 (6.0)	10–32	30.7 (7.0)	20–45	27.8 (7.1)	10–45
C7–Th5 flexion mobility (mm)	27 (5)	18–40	27 (5)	20–38	27 (5)	18–40
Schober (spine in flexion; mm)	41 (10)	24–60	45 (11)	27–65	43 (11)	24–65
Schober (spine in extension; mm)^c^	22 (11)	2–45	23 (14)	−7 – 45	22 (13)	−7 – 45
Thoracic flexion mobility, sitting (°)						
Upper (Th1–6)	14.3 (4.2)	5–23	13.5 (6.5)	3–25	13.9 (5.4)	3–25
Lower (Th6–12)	12.4 (4.8)	4–21	15.4 (9.6)	4–40	13.9 (7.6)	4–40
Upper and lower (Th1–12)	26.1 (5.0)	15–32	28.9 (10.9)	14–48	27.5 (8.5)	14–48
Thoracic extension mobility, sitting (°)						
Upper (Th1–6)^d^	12.1 (7.4)	−4 – 26	12.1 (12.1)	−3 – 50	12.1 (9.8)	−4 – 50
Lower (Th6–12)^d^	5.2 (6.1)	−5 – 19	5.1 (6.4)	−10 – 19	5.2 (6.1)	−10 – 19
Upper and lower (Th1–12)^d^	17.3 (10.6)	3–36	17.2 (16.6)	−4 – 69	17.2 (13.7)	−4 – 69

*SD* Standard deviation; *Th* Thoracic; *df* Degree of freedom

^a^ The difference between sitting and standing measurements were evaluated with paired-samples t-test

^b^ Negative values represent thoracic extension

^c^ Negative values represent increased thoracic kyphosis (i.e. flexion) while trying to extend the thoracic spine

^d^ Negative values represent inability to extend thoracic spine (i.e. thoracic spine remains in flexion while trying to extend it)

### Inter-rater reliability

The inspection of thoracic spine posture and the manual assessment of mobility had very low reliability in inter-rater evaluation. In contrast, inter-rater reliability was very strong for the inclinometer measurements of upper thoracic spine posture in sitting and standing positions (ICC 0.85 and 0.81, respectively) and whole thoracic spine posture in a standing position (ICC 0.82). Other measurements did not reach to our target reliability of higher than 0.80 value. Inter-rater reliability was strong for the tape measurements in a neutral position and the thoracic spine Schober flexion value (ICC 0.74, both). Extension mobility measured with the inclinometer had strong reliability in the upper and whole thoracic spine (ICC 0.61 and 0.62, respectively), while the other mobility measurements were moderate at best. All the ICC values and measurement errors for inter-rater reliability are shown in Tables 5 and 6.

**Table 5:** Inter- and intra-rater reliability (Intra-class correlation coefficients, ICC) with 95% confidence interval (CI) and Cronbach’s alpha of thoracic spine posture and mobility measurements

Variable	Inter-rater (*n* = 32)			Intra-rater (*n* = 32)		
	ICC	CI 95%	Cronbach α	ICC	CI 95%	Cronbach α
Posture inspection while standing^a^	0.28	(−0.55–0.67)	0.28	0.78	(0.56–0.89)	0.78
Posture inspection while sitting^a^	0.23	(−0.58–0.62)	0.23	0.87	(0.73–0.94)	0.87
Segmental mobility into flexion^b^	−0.07	(−1.20–0.48)	−0.07	0.79	(0.56–0.90)	0.79
Segmental mobility into extension^b^	−0.38	(−1.84–0.33)	− 0.38	0.80	(0.59–0.90)	0.80
Posterior to anterior pressure^b^	−0.17	(−1.39–0.43)	− 0.17	0.69	(0.37–0.85)	0.69
Inclination of Th1–6 while standing (°)	0.81	(0.60–0.91)	0.82	0.86	(0.72–0.93)	0.86
Inclination of Th6–12 while standing (°)	0.69	(0.37–0.85)	0.69	0.70	(0.39–0.85)	0.72
Inclination of Th1–12 while standing (°)	0.82	(0.64–0.91)	0.82	0.83	(0.66–0.92)	0.83
Inclination of Th1–6 while sitting (°)	0.85	(0.67–0.93)	0.86	0.84	(0.67–0.92)	0.84
Inclination of Th6–12 while sitting (°)	0.60	(0.18–0.80)	0.60	0.80	(0.60–0.90)	0.80
Inclination of Th1–12 while sitting (°)	0.70	(0.37–0.85)	0.69	0.74	(0.47–0.87)	0.74
C7-Th5 flexion mobility (tape, mm)	0.28	(− 0.37–0.63)	0.30	0.66	(0.30–0.83)	0.65
Schober (spine in neutral, mm)	0.74	(0.47–0.87)	0.74	0.86	(0.72–0.93)	0.86
Schober (spine in flexion, mm)	0.74	(0.48–0.87)	0.75	0.72	(0.43–0.86)	0.73
Schober (spine in extensio, mm)	0.29	(−0.48–0.66)	0.28	0.30	(− 0.42–0.66)	0.30
Flexion mobility of Th1–6 while sitting (°)	0.19	(−0.67–0.60)	0.19	0.64	(0.26–0.82)	0.64
Flexion mobility of Th6–12 while sitting (°)	0.52	(0.02–0.77)	0.52	0.49	(−0.05–0.75)	0.49
Flexion mobility of Th1–12 while sitting (°)	0.48	(−0.06–0.75)	0.48	0.67	(0.32–0.84)	0.67
Extension mobility of Th1–6 while sitting (°)	0.61	(0.20–0.81)	0.61	0.35	(−0.33–0.68)	0.35
Extension mobility of Th6–12 while sitting (°)	0.58	(0.15–0.80)	0.58	0.30	(−0.43–0.66)	0.30
Extension mobility of Th1–12 while sitting (°)	0.62	(0.22–0.81)	0.62	0.46	(−0.10–0.74)	0.46

^a^ Evaluated in three categories: decreased thoracic kyphosis (flat back), normal kyphosis or hyperkyphosis

^b^ Sum of all thoracic spine motion segments recorded as hypomobile

**Table 6:** Inter- and intra-rater reproducibility as standard error of measurement (SEM), mean of differences (MD), beta value of linear regression on Bland-Altman analyses (B) and coefficient of repeatability (CR) of thoracic spine posture and mobility measurements

Variable	Inter-rater (*n* = 32)				Intra-rater (*n* = 32)			
	SEM	MD	B^c^	CR	SEM	MD	B^c^	CR
Posture inspection while standing^a^	0.56	−1.00	−0.21	1.56	0.31	0.06	− 0.01	0.86
Posture inspection while sitting^a^	0.61	−0.69	−0.56	1.68	0.25	−0.03	− 0.08	0.69
Segmental mobility into flexion^b^	2.62	0.88	−0.69	7.27	1.16	−0.53	0.34	3.22
Segmental mobility into extension^b^	3.26	1.91	−0.13	9.03	1.24	−0.50	−0.02	3.44
Posterior to anterior pressure^b^	1.89	1.44	−0.58	5.23	1.15	−0.22	0.28	3.20
Inclination of Th1–6 while standing (°)	2.43	−2.0	−0.26	6.73	2.10	−0.81	−0.15	5.80
Inclination of Th6–12 while standing (°)	2.67	0.47	−0.28	7.40	2.60	1.84	−0.29	7.20
Inclination of Th1–12 while standing (°)	3.18	−1.34	−0.25	8.81	3.09	1.34	−0.10	8.57
Inclination of Th1–6 while sitting (°)	2.66	−2.16	−0.15	7.37	2.76	−0.94	0.02	7.65
Inclination of Th6–12 while sitting (°)	3.92	1.28	−0.19	10.86	2.77	−0.97	−0.11	7.68
Inclination of Th1–12 while sitting (°)	3.87	−0.53	−0.17	10.72	3.62	−1.34	−0.18	10.03
C7-Th5 flexion mobility (tape, mm)	4.17	3.59	−0.75	11.59	2.87	0.72	−0.15	7.95
Schober (spine in neutral, mm)	16.08	−4.47	0.08	44.55	11.80	2.47	0.12	32.69
Schober (spine in flexion, mm)	5.47	2.41	−0.20	15.15	5.67	2.22	0.26	15.71
Schober (spine in extensio, mm)	10.53	−4.75	0.21	29.17	10.88	0.13	−0.03	30.13
Flexion mobility of Th1–6 while sitting (°)	4.86	1.69	0.88	13.46	3.24	1.06	0.17	8.97
Flexion mobility of Th6–12 while sitting (°)	5.27	4.22	0.94	14.59	5.43	1.91	0.62	15.03
Flexion mobility of Th1–12 while sitting (°)	6.13	−2.88	0.84	16.98	4.88	2.41	0.33	13.53
Extension mobility of Th1–6 while sitting (°)	6.12	0.59	0.04	16.95	7.90	−1.47	0.46	20.89
Extension mobility of Th6–12 while sitting (°)	3.95	5.97	0.29	10.95	5.10	−1.38	−0.15	14.14
Extension mobility of Th1–12 while sitting (°)	8.45	5.16	0.17	23.39	10.07	−1.38	0.42	27.89

^a^ Evaluated in three categories: decreased thoracic kyphosis (flat back), normal kyphosis or hyperkyphosis

^b^ Sum of all thoracic spine motion segments recorded as hypomobile

^c^ The Bland Altman plots for each item are presented in the additional electronic files of this study; none of the linear regression for proportional bias were statistically significant

### Intra-rater reliability

The inspection of the thoracic spine posture in standing and in sitting had strong or very strong reliability (ICC 0.78 and 0.87, respectively) in intra-rater assessment. The segmental mobility assessment into extension had the highest values (ICC 0.80); however, other manual intra-rater assessments also had strong reliability values (ICC 0.69–0.73). The inclination measurements of the thoracic spine posture had strong or very strong intra-rater reliability. Very strongly reliable part of the spine to measure with inclinometer was upper thoracic spine (0.86 and 0.84 in standing and sitting, respectively) whereas lower thoracic spine in standing and whole thoracic spine in sitting had only strong reliability (0.70 and 0.74, respectively) (Table 5). Moreover, the neutral thoracic spine posture assessed with a measuring tape had very strong reliability (0.86), while the thoracic spine Schober flexion and the C7-T5 flexion mobility had strong intra-rater reliability (ICC 0.72 and 0.66, respectively). The maximum thoracic spine extension and flexion measured with the inclinometer had very weak to moderate and weak to strong reliability, respectively, on an intra-rater analyses. All the ICC values are presented for intra-rater reliability in Table 5, and the measurement errors are presented in Table 6. Bland-Altman plots and 95% limits of agreements are shown in additional electronic file. No proportional biases were detected when linear regression was performed for Bland-Altman plots.

## Discussion

In the current study, the intra-rater reliability of thoracic spine posture inspection and manual assessments had at least strong reliability in the subjects with thoracic spine pain. Posture measurements with an inclinometer and Schober flexion with a tape had at least strong inter- and intra-rater reliability.

To the best of our knowledge, this study was the first to evaluate the reliability on wide range of posture and mobility measurements used in daily clinical practice for subjects with thoracic spine pain in a clinical setting. The major weakness of the pre-existing studies is the insufficient statistical analysis, as most of these studies reported only correlation coefficients or ICCs, and none reported the limits of agreement or other measurement error values. Ng et al. [35] reported the coefficient of variation to somewhat explain the dispersion around the mean of ICC, and Tousignant et al. [36] reported 95% confidence intervals of ICC. Both of these studies reported at least moderate intra-rater reliability of the measurements of cervical and lumbar spine mobility with an inclinometer, but both were performed with asymptomatic subjects. More recently, one systematic review evaluated the reliability and validity of the thoracic and lumbar spine posture measurement reliability and reported that digital and manual inclinometer can be reliable method to evaluate spinal posture [21] similarly as we did. However, they did not report inclinometers to be the best evaluation method as the validity of these measure devices has not been studied thoroughly. One study has been published on same digital inclinometer that we used in the current study with very strong reliability (Cronbach’s alpha 0.82–0.86), but the study was performed with asymptomatic subjects [29].

### Assessment of the thoracic spine posture

An earlier study of 88 healthy subjects by Griegel-Morris et al. [37] found the intra- and inter-rater reliability of thoracic spine posture inspection to be 0.83 and 0.61, respectively. Moreover, in another study, the inter-rater reliability of the observed thoracic spine kyphosis was found to be between 0.58 and 0.90 in subjects with cervical spine pain [38]. In our study, the inter-rater reliability was strong (ICC = 0.78) in a standing position and very strong (ICC = 0.87) in a sitting position in subjects with thoracic spine pain. Based on our result, we would recommend to be cautious when comparing posture evaluation between therapists as the inter-rater reliability was weak in sitting and standing positions (ICC = 0.23 and 0.28, respectively). In contrast to the earlier studies by Griegel-Morris et al [37] and Cleland et al [38], we did not find good inter-rater reliability in posture inspection, however, in line with the Griegel-Morris et al [37], the intra-rater reliability was higher than inter-rater reliability and reliability is acceptable for clinical use. Moreover, we found that reliability is even higher in sitting than standing, as earlier studies have only evaluated the posture inspection on standing [37, 38].

### Manual assessment of the thoracic spine mobility

The manual evaluation of thoracic spine mobility has been criticized due to its low reliability, especially between observers [19, 38–41]. However, several studies have found the intra-rater reliability of thoracic spine mobility assessed manually to be at least moderate in symptomatic and asymptomatic subjects [19, 42–44]. However, the skill of finding the desired spinal segment and identifying it similarly between observers seems to be difficult and unreliable [45]; there seems to be a great difference between observers [40], and none of the palpation methods seem to be superior to evaluating thoracic spine mobility manually [24]. Heiderscheitet et al [46] reposted in the asymptomatic subjects the intra-rater reliability of PA pressure of the thoracic spine has strong reliability (kappa = 0.61–0.75), whereas in interrater reliability kappa value was lower (0.59). Our results are in line with Heiderscheitet et al [46], as inter-rater reliability in our study was very weak for PA pressure, whereas intra-rater reliability was strong. Walker et al [47] reported segmental mobility test of the thoracic spine in sitting to have weak inter-rater reliability (kappa = 0.36), which is in line with our results. However, we found intra-rater reliability to be strong or even very strong. The challenge in measurement is the accuracy of the palpation of thoracic spine segments, which has been reported to be poor in earlier studies [19]. This inaccuracy may also explain the low reliability of many inter-rater measurements, as in our study.

Earlier studies have stated that the finding of hypomobility or dysfunction in the symptomatic area of the spine seems to be more relevant than numbering the segment similarly between observers [23, 42]. Therefore, the symptoms on the hypomobile area of the spine are clinically relevant when thoracic spine manipulation is the treatment of choice, as manipulation may increase thoracic spine mobility [48–51]. Therefore, manual assessment is important as part of the subject examination, and it can be quite reliable, especially when the same physical therapist performs the examination.

### Assessment of the thoracic spine posture and mobility with inclinometer and tape measure

In the current study, the reliability of the posture measurements with an inclinometer were strong or very strong in both inter-rater and intra-rater evaluations. As palpation or subjective observation is only minimally involved in inclinometer measurement, it is understandable that this method of postural assessment would be more reliable. However, as in other measurements, the intra-rater reliability was better than inter-rater reliability. Czaprowski et al [29] have earlier reported that thoracic spine kyphosis can be measured with high reliability (Cronbach α > 0.8) in standing among asymptomatic subjects with as little as 3 degrees of measurement error. We found similar measurement error in our symptomatic subjects. Several other methods of measurements for the thoracic spine posture have been used and recently, their reliability and validity has been reported in the review. Most of the included measurement devices had very strong reliability and only a few were studied on their validity [21].

In an earlier study [22], the reliability of lumbar flexion and extension mobility measured with the modified Schober test was strong (ICC 0.72 and 0.76, respectively). In the current study, posture measured with a tape had strong reliability for inter-rater and very strong reliability for intra-rater measurements. However, Schober extension was not reliable in intra-rater or inter-rater measurements. One possible reason is the low degree of thoracic spine extension, which may make it challenging to obtain similar results in different measurements. Thus, we did not find good reliability of thoracic extension mobility measurements using a tape measure. We did not find any studies investigating the flexion or extension of the thoracic spine with tape measurement.

There are several studies investigating the reliability of cervical [36, 52] and lumbar spine [35, 53, 54] mobility measurement devices among asymptomatic adults. Williams et al [22] reported lumbar spine flexion and extension mobility inter-rater reliability with double inclinometer (ICC 0.60 and 0.48, respectively) and found that measuring flexion is more reliable than extension. In the current study, the thoracic spine extension mobility was most reliably measured using an inclinometer (whole and upper thoracic spine) between two therapists, while flexion mobility (whole and upper thoracic spine) was most reliable between consecutive days by the same therapist. Moreover, the reliability of the lower thoracic spine extension using an inclinometer was weak, perhaps due to the activation of spinal extensors, which could have made it difficult to place the inclinometer in firm contact with the spinous process because the contact pillar of the inclinometer was wider than the spinous process. We did not find any earlier reliability studies on thoracic spine mobility using a digital inclinometer. However, a few studies have been done for spinal mouse with at least strong inter- and intra-rater reliability (ICC 0.67–0.95 and ICC 0.67–0.88, respectively) [55, 56].

The range of normal kyphosis of the thoracic spine is 20 to 50 degrees [21]. In the current study, the mean thoracic kyphosis was within normal limits; however, there were also female subjects with hypokyphosis. In the present study, two thirds of the thoracic spine kyphosis was found in upper thoracic spine which is in line with earlier study by Czaprowski et al [29], however, they reported even higher proportion of thoracic spine kyphosis originating from upper thoracic spine. In asymptomatic males, extension has been reported to be more than 10 degrees, and one-third of the extension occurs in the upper thoracic spine [9]. We found the total extension of the thoracic spine to be 17° in a sitting position. Contrary to Edmonston et al. [9], our subjects had two-thirds of the thoracic spine extension arising from the upper thoracic spine, while one-third was from the lower thoracic spine. One of the influencing factors may be the sitting posture of the subjects during measurements in the present study. Moreover, Edmonston et al [9] measured the range of motion into extension while flexing the upper limb. In the two studies in which the spinal mouse was used, total amount of thoracic spine flexion were 17 and 25 degrees in asymptomatic young elite cross-country skiers [57] and asymptomatic middle-aged subjects [56], respectively. In the present study, the mean age of the subjects was much closer to middle-aged than under 20-year-old and, therefore, it is more likely that our subjects should have similarities in the thoracic flexion mobility with subjects in the study of Mannion et al [56]. However, our subjects had extension of the thoracic spine similar to the young elite cross-country skiers (17 degrees) [57] as in the elder study population in the study by Mannion et al [56] the mean extension was reported to be only a few degrees.

### Strengths and limitations of the study

The strength of this study is its ease of accessibility and inexpensive measurement devices, the reliability of which was measured in symptomatic subjects. The reliability on consecutive days was measured; however, week-to-week reliability was not studied, which is a limitation of the present study, as physical therapy sessions are often scheduled once a week. The second limitation of the study is that two physical therapists did the assessments only 10-min apart from each other and therefore some changes in mobility of the thoracic spine may have occurred. Thirdly, one physical therapist (JT) did the assessments on consecutive days and, therefore, there might have been a possibility to remember the initial recordings. However, this was not the case as physical therapist was requested to write down the previous results prior the second assessment and he was not able to do that. One of the strengths of this study is that physical therapists did have different background and work experience in years. Although physical therapists agreed on manual examination protocol and scale prior to the study, both of them did the evaluation based on their own experience and post graduate training on spinal mobility. The inter-rater reliability may have been different if the physical therapists had had similar experience and post graduate background in manual therapy. This should be evaluated in future studies whether the similar manual therapy training would improve the inter-rater reliability of manual mobility examination of thoracic spine. Finally, one strength of the current study is that we have reported wide range of reliability data such as ICC with 95% confidence intervals, Cronbach’s alpha, SEM, mean of differences, CR and Bland-Altman plots with their 95% limits of agreement and linear regression for proportional bias. Reporting the CR can help a clinician to evaluate clinically meaningful change in patients’ as it quantifies the absolute reliability measurement error using the same units as the measurement device itself. CR is directly related to the 95% limits of agreement by Bland-Altman plots on same subject and it takes random and systematic errors into account. Thus, the change is real with 95% confident in subjects’ measurements, if it is higher than CR [34].

## Conclusion

In subjects with thoracic spine pain, daily intra-rater reliability was strong or very strong for inspection of the posture and manual mobility evaluation methods mobility of the thoracic spine. Moreover, inter-rater reliability was strong or very strong for the evaluation of posture with inclinometer and flexion mobility using a tape measure. Both intra-rater and inter-rater reliability were lower in evaluating the thoracic spine mobility with inclinometer.

## Supplementary information

**Additional file 1.** Additional Figure. 1–7. Bland–Altman plot for agreement of posture inspection in standing (1A and 1B), posture inspection in sitting (2A and 2B), segmental mobility into flexion (3A and 3B), segmental mobility into extension (4A and 4B), posterior to anterior pressure (5A and 5B), inclination of T1–6 in standing (6A and 6B), inclination of T6–12 in standing (7A and 7B), inclination of T1–12 in standing (8A and 8B), inclination of T1–6 in sitting (9A and 9B), inclination of T6–12 in sitting (10A and 10B), inclination of T1–12 in sitting (11A and 11B), C7–T5 flexion mobility (12A and 12B), Schober in neutral (13A and 13B), Schober in flexion (14A and 14B), Schober in extension (15A and 15B), flexion mobility of the T1–6 in sitting (16A and 16B), flexion mobility of the T6–12 in sitting (17A and 17B), flexion mobility of the T1–12 in sitting (18A and 18B), extension mobility of the T1–6 in sitting (19A and 19B), extension mobility of the T6–12 in sitting (20A and 20B) and extension mobility of the T1–12 in sitting (21A and 21B) between raters (A) and within rater (B). The red lines depict the mean difference between raters and dotted green lines depict 95% limits of agreement in the Bland–Altman plot.

## Data Availability

The datasets used and/or analysed during the current study are available from the corresponding author on reasonable request.

## Abbreviations

VAS: Visual analogue scale
ICC: Intra-class coefficiency
R-MDQ: Roland-Morris disability questionnaire
TSPD: Thoracic spine pain and disability
PA: Posterior-anterior
SEM: Standard error of measurement
SD: Standard deviation
CR: Coefficient of repeatability
