# Supplementary figures and images for: Intra- and inter-rater reliability of thoracic spine mobility and posture assessments in subjects with thoracic spine pain
Source: BMC Musculoskelet Disord. 2020 Aug 10;21:529. doi: 10.1186/s12891-020-03551-4 (PMC7418198; doi:10.1186/s12891-020-03551-4)

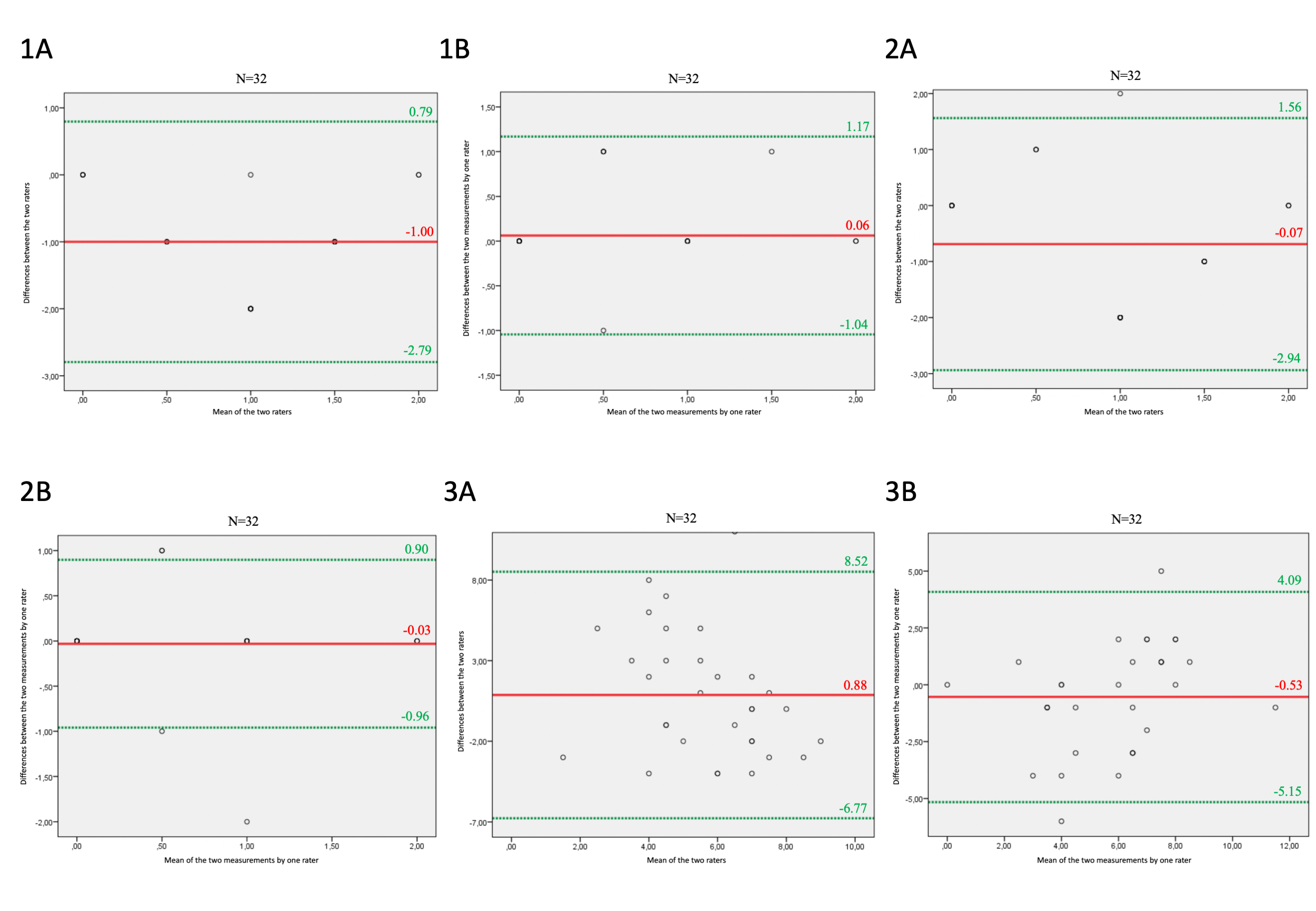

Supplement: Supplementary file 1 — Additional file 1. Additional Figure. 1–7. Bland–Altman plot for agreement of posture inspection in standing (1A and 1B), posture inspection in sitting (2A and 2B), segmental mobility into flexion (3A and 3B), segmental mobility into extension (4A and 4B), posterior to anterior pressure (5A and 5B), inclination of T1–6 in standing (6A and 6B), inclination of T6–12 in standing (7A and 7B), inclination of T1–12 in standing (8A and 8B), inclination of T1–6 in sitting (9A and 9B), inclination of T6–12 in sitting (10A and 10B), inclination of T1–12 in sitting (11A and 11B), C7–T5 flexion mobility (12A and 12B), Schober in neutral (13A and 13B), Schober in flexion (14A and 14B), Schober in extension (15A and 15B), flexion mobility of the T1–6 in sitting (16A and 16B), flexion mobility of the T6–12 in sitting (17A and 17B), flexion mobility of the T1–12 in sitting (18A and 18B), extension mobility of the T1–6 in sitting (19A and 19B), extension mobility of the T6–12 in sitting (20A and 20B) and extension mobility of the T1–12 in sitting (21A and 21B) between raters (A) and within rater (B). The red lines depict the mean difference between raters and dotted green lines depict 95% limits of agreement in the Bland–Altman plot. [file 12891_2020_3551_MOESM1_ESM.zip › Additional figure 1R5.png]

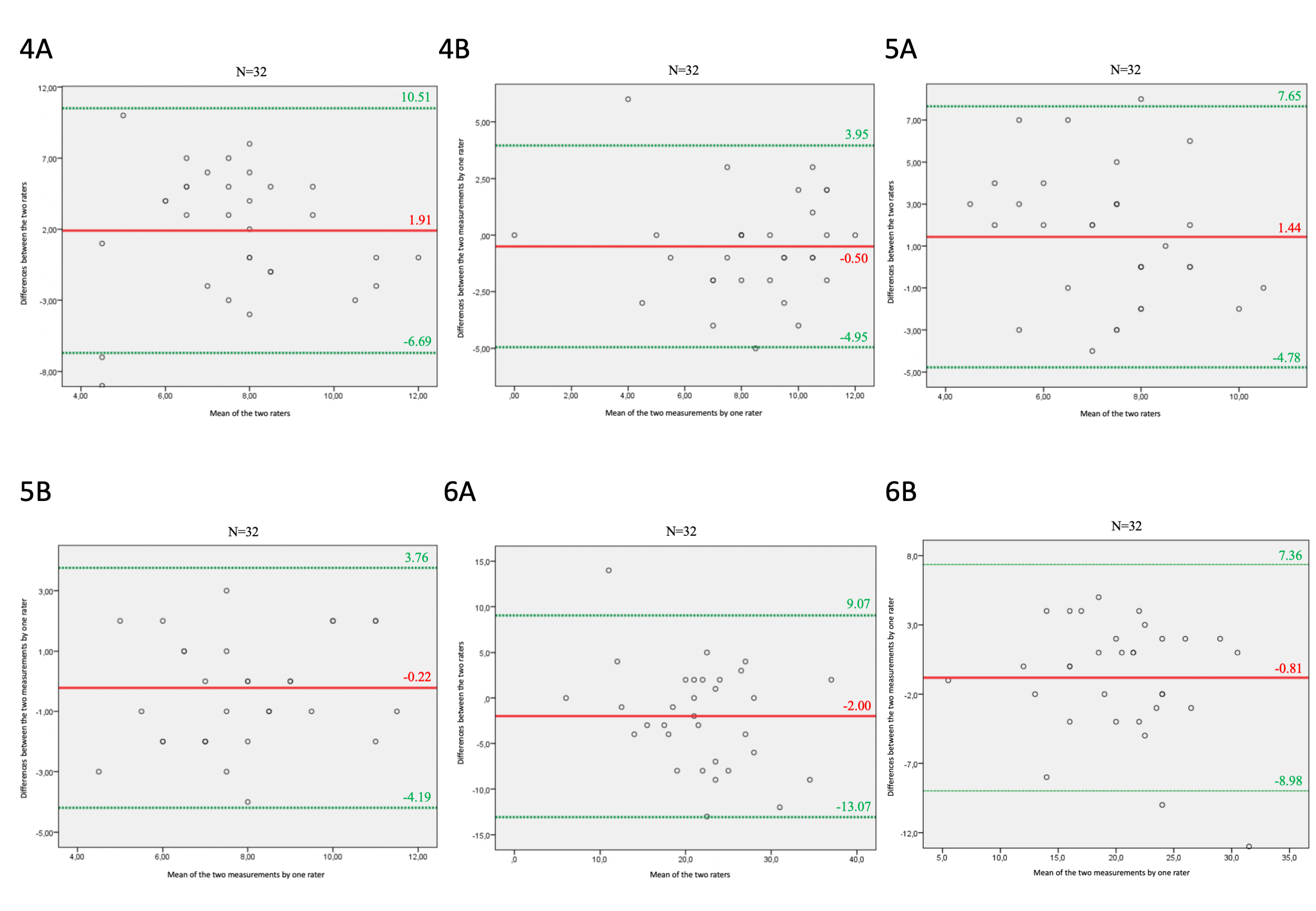

Supplement: Supplementary file 1 — Additional file 1. Additional Figure. 1–7. Bland–Altman plot for agreement of posture inspection in standing (1A and 1B), posture inspection in sitting (2A and 2B), segmental mobility into flexion (3A and 3B), segmental mobility into extension (4A and 4B), posterior to anterior pressure (5A and 5B), inclination of T1–6 in standing (6A and 6B), inclination of T6–12 in standing (7A and 7B), inclination of T1–12 in standing (8A and 8B), inclination of T1–6 in sitting (9A and 9B), inclination of T6–12 in sitting (10A and 10B), inclination of T1–12 in sitting (11A and 11B), C7–T5 flexion mobility (12A and 12B), Schober in neutral (13A and 13B), Schober in flexion (14A and 14B), Schober in extension (15A and 15B), flexion mobility of the T1–6 in sitting (16A and 16B), flexion mobility of the T6–12 in sitting (17A and 17B), flexion mobility of the T1–12 in sitting (18A and 18B), extension mobility of the T1–6 in sitting (19A and 19B), extension mobility of the T6–12 in sitting (20A and 20B) and extension mobility of the T1–12 in sitting (21A and 21B) between raters (A) and within rater (B). The red lines depict the mean difference between raters and dotted green lines depict 95% limits of agreement in the Bland–Altman plot. [file 12891_2020_3551_MOESM1_ESM.zip › Additional figure 2R5.png]

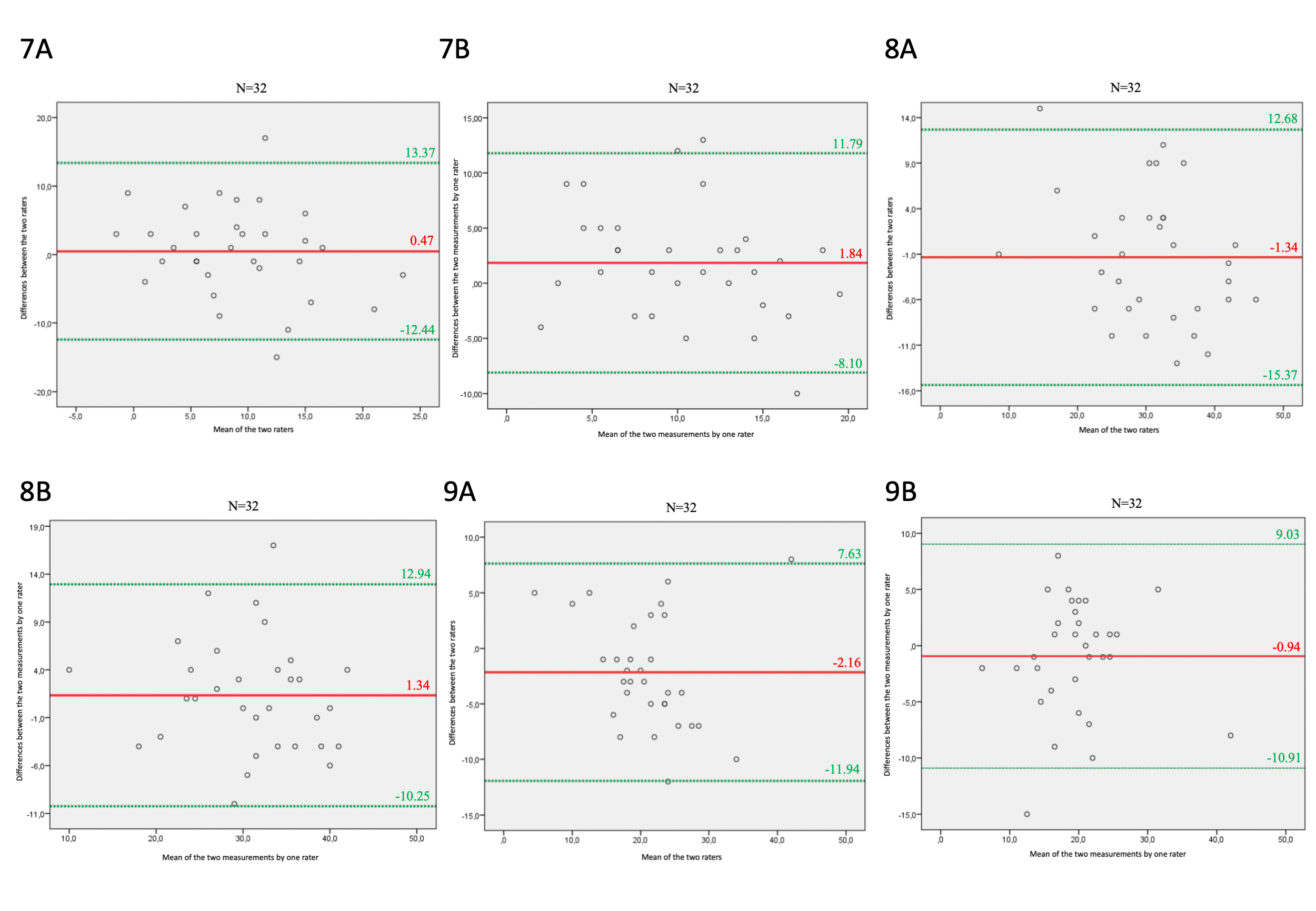

Supplement: Supplementary file 1 — Additional file 1. Additional Figure. 1–7. Bland–Altman plot for agreement of posture inspection in standing (1A and 1B), posture inspection in sitting (2A and 2B), segmental mobility into flexion (3A and 3B), segmental mobility into extension (4A and 4B), posterior to anterior pressure (5A and 5B), inclination of T1–6 in standing (6A and 6B), inclination of T6–12 in standing (7A and 7B), inclination of T1–12 in standing (8A and 8B), inclination of T1–6 in sitting (9A and 9B), inclination of T6–12 in sitting (10A and 10B), inclination of T1–12 in sitting (11A and 11B), C7–T5 flexion mobility (12A and 12B), Schober in neutral (13A and 13B), Schober in flexion (14A and 14B), Schober in extension (15A and 15B), flexion mobility of the T1–6 in sitting (16A and 16B), flexion mobility of the T6–12 in sitting (17A and 17B), flexion mobility of the T1–12 in sitting (18A and 18B), extension mobility of the T1–6 in sitting (19A and 19B), extension mobility of the T6–12 in sitting (20A and 20B) and extension mobility of the T1–12 in sitting (21A and 21B) between raters (A) and within rater (B). The red lines depict the mean difference between raters and dotted green lines depict 95% limits of agreement in the Bland–Altman plot. [file 12891_2020_3551_MOESM1_ESM.zip › Additional figure 3R5.png]

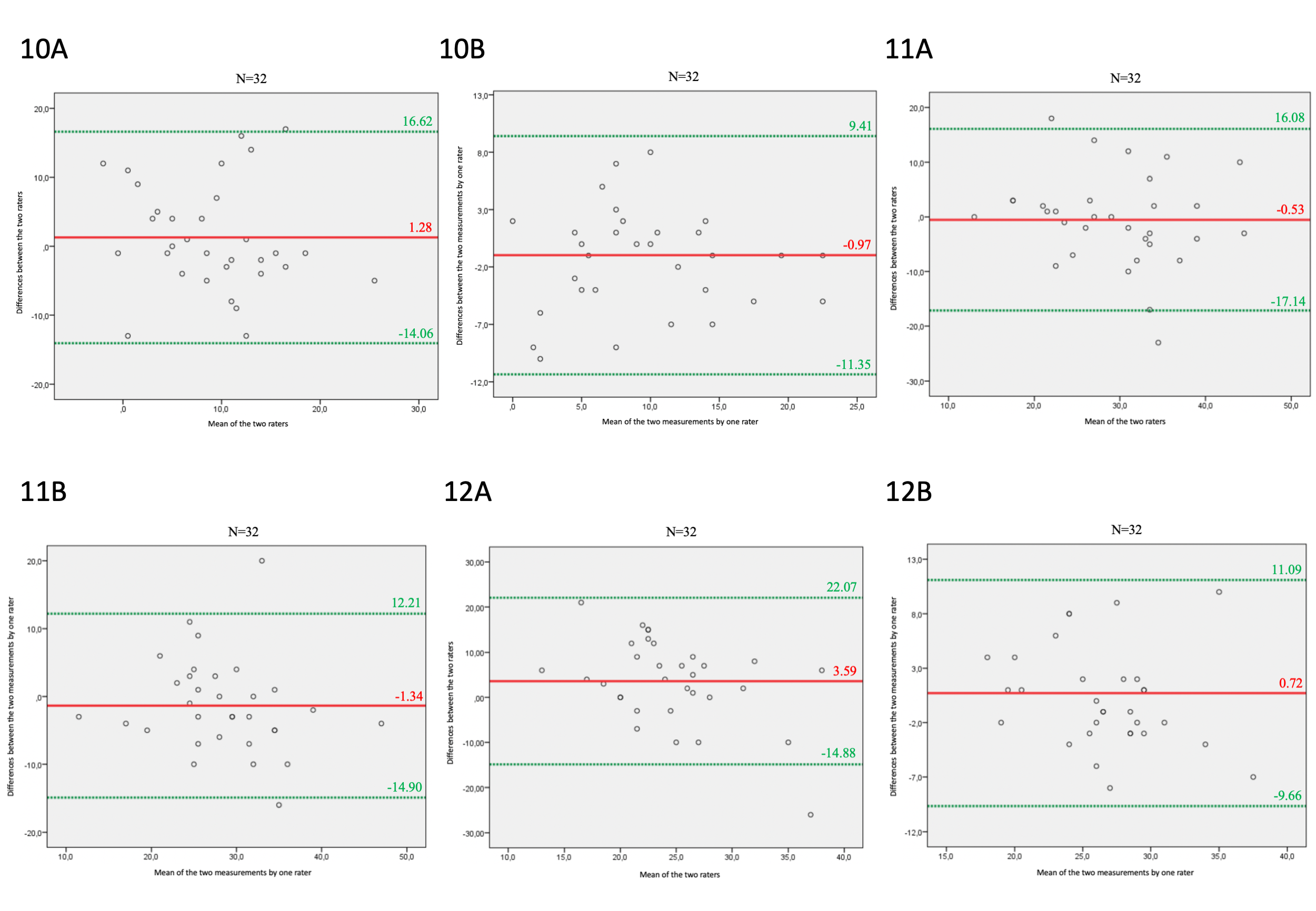

Supplement: Supplementary file 1 — Additional file 1. Additional Figure. 1–7. Bland–Altman plot for agreement of posture inspection in standing (1A and 1B), posture inspection in sitting (2A and 2B), segmental mobility into flexion (3A and 3B), segmental mobility into extension (4A and 4B), posterior to anterior pressure (5A and 5B), inclination of T1–6 in standing (6A and 6B), inclination of T6–12 in standing (7A and 7B), inclination of T1–12 in standing (8A and 8B), inclination of T1–6 in sitting (9A and 9B), inclination of T6–12 in sitting (10A and 10B), inclination of T1–12 in sitting (11A and 11B), C7–T5 flexion mobility (12A and 12B), Schober in neutral (13A and 13B), Schober in flexion (14A and 14B), Schober in extension (15A and 15B), flexion mobility of the T1–6 in sitting (16A and 16B), flexion mobility of the T6–12 in sitting (17A and 17B), flexion mobility of the T1–12 in sitting (18A and 18B), extension mobility of the T1–6 in sitting (19A and 19B), extension mobility of the T6–12 in sitting (20A and 20B) and extension mobility of the T1–12 in sitting (21A and 21B) between raters (A) and within rater (B). The red lines depict the mean difference between raters and dotted green lines depict 95% limits of agreement in the Bland–Altman plot. [file 12891_2020_3551_MOESM1_ESM.zip › Additional figure 4R5.png]

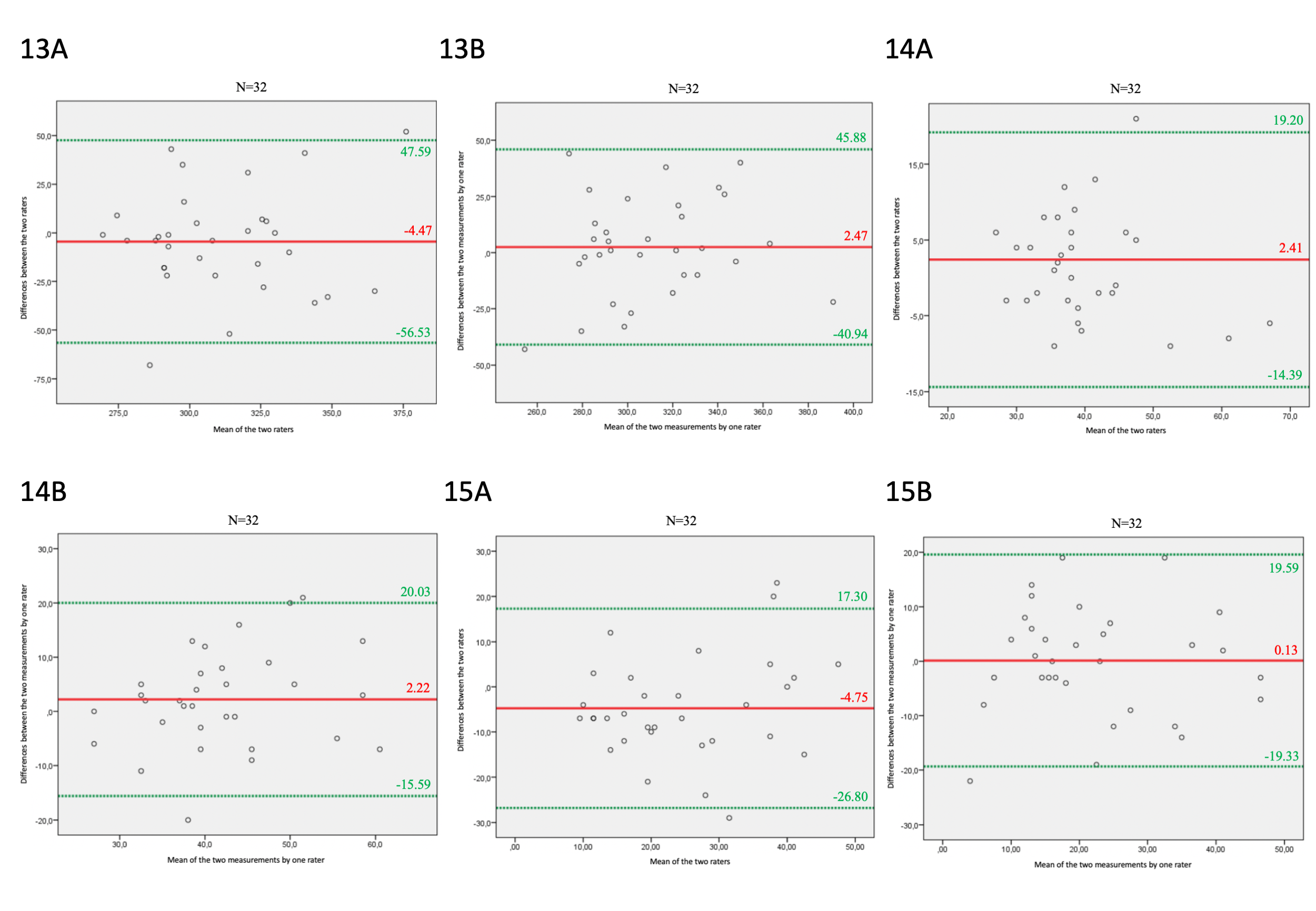

Supplement: Supplementary file 1 — Additional file 1. Additional Figure. 1–7. Bland–Altman plot for agreement of posture inspection in standing (1A and 1B), posture inspection in sitting (2A and 2B), segmental mobility into flexion (3A and 3B), segmental mobility into extension (4A and 4B), posterior to anterior pressure (5A and 5B), inclination of T1–6 in standing (6A and 6B), inclination of T6–12 in standing (7A and 7B), inclination of T1–12 in standing (8A and 8B), inclination of T1–6 in sitting (9A and 9B), inclination of T6–12 in sitting (10A and 10B), inclination of T1–12 in sitting (11A and 11B), C7–T5 flexion mobility (12A and 12B), Schober in neutral (13A and 13B), Schober in flexion (14A and 14B), Schober in extension (15A and 15B), flexion mobility of the T1–6 in sitting (16A and 16B), flexion mobility of the T6–12 in sitting (17A and 17B), flexion mobility of the T1–12 in sitting (18A and 18B), extension mobility of the T1–6 in sitting (19A and 19B), extension mobility of the T6–12 in sitting (20A and 20B) and extension mobility of the T1–12 in sitting (21A and 21B) between raters (A) and within rater (B). The red lines depict the mean difference between raters and dotted green lines depict 95% limits of agreement in the Bland–Altman plot. [file 12891_2020_3551_MOESM1_ESM.zip › Additional figure 5R5.png]

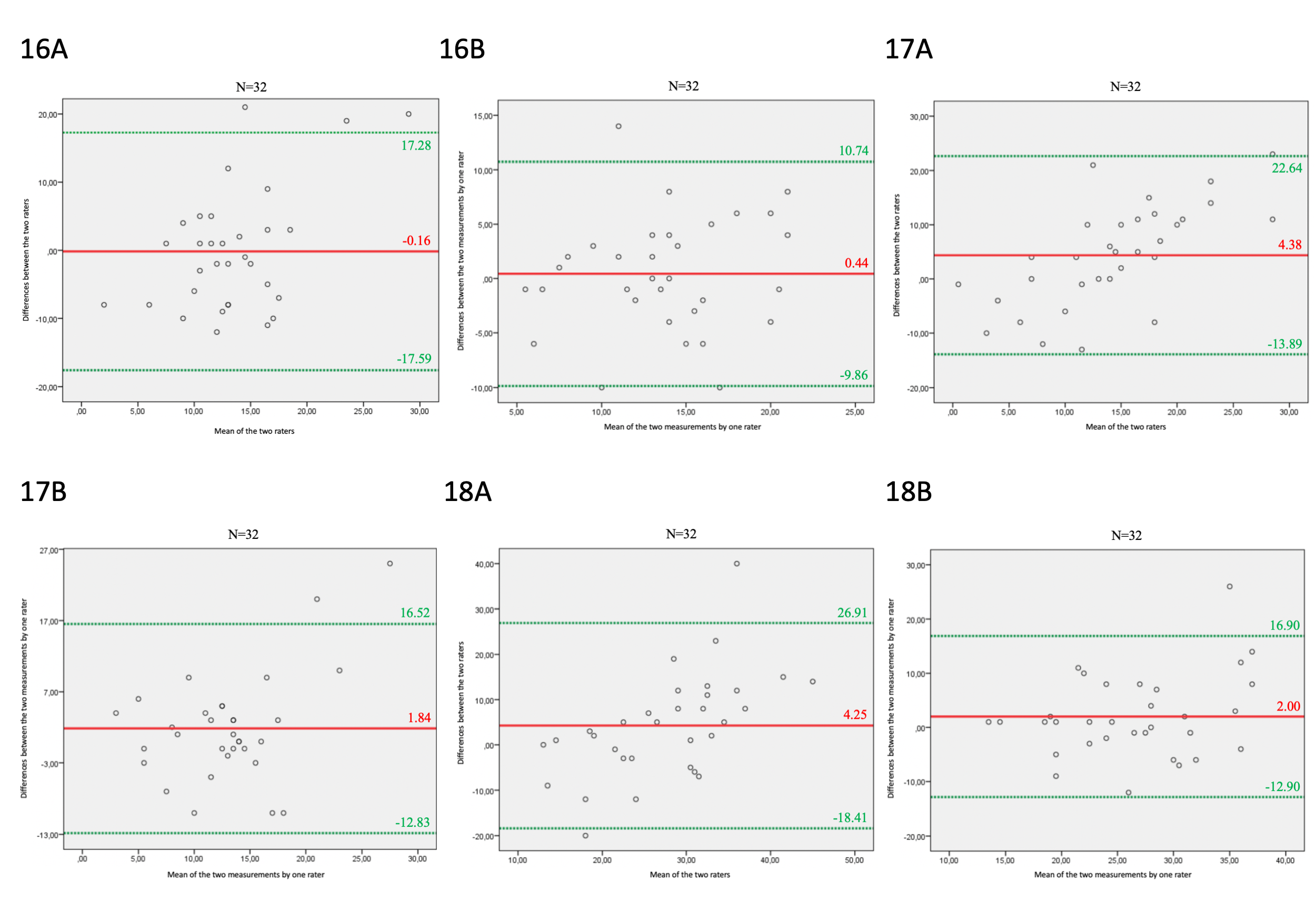

Supplement: Supplementary file 1 — Additional file 1. Additional Figure. 1–7. Bland–Altman plot for agreement of posture inspection in standing (1A and 1B), posture inspection in sitting (2A and 2B), segmental mobility into flexion (3A and 3B), segmental mobility into extension (4A and 4B), posterior to anterior pressure (5A and 5B), inclination of T1–6 in standing (6A and 6B), inclination of T6–12 in standing (7A and 7B), inclination of T1–12 in standing (8A and 8B), inclination of T1–6 in sitting (9A and 9B), inclination of T6–12 in sitting (10A and 10B), inclination of T1–12 in sitting (11A and 11B), C7–T5 flexion mobility (12A and 12B), Schober in neutral (13A and 13B), Schober in flexion (14A and 14B), Schober in extension (15A and 15B), flexion mobility of the T1–6 in sitting (16A and 16B), flexion mobility of the T6–12 in sitting (17A and 17B), flexion mobility of the T1–12 in sitting (18A and 18B), extension mobility of the T1–6 in sitting (19A and 19B), extension mobility of the T6–12 in sitting (20A and 20B) and extension mobility of the T1–12 in sitting (21A and 21B) between raters (A) and within rater (B). The red lines depict the mean difference between raters and dotted green lines depict 95% limits of agreement in the Bland–Altman plot. [file 12891_2020_3551_MOESM1_ESM.zip › Additional figure 6R5.png]

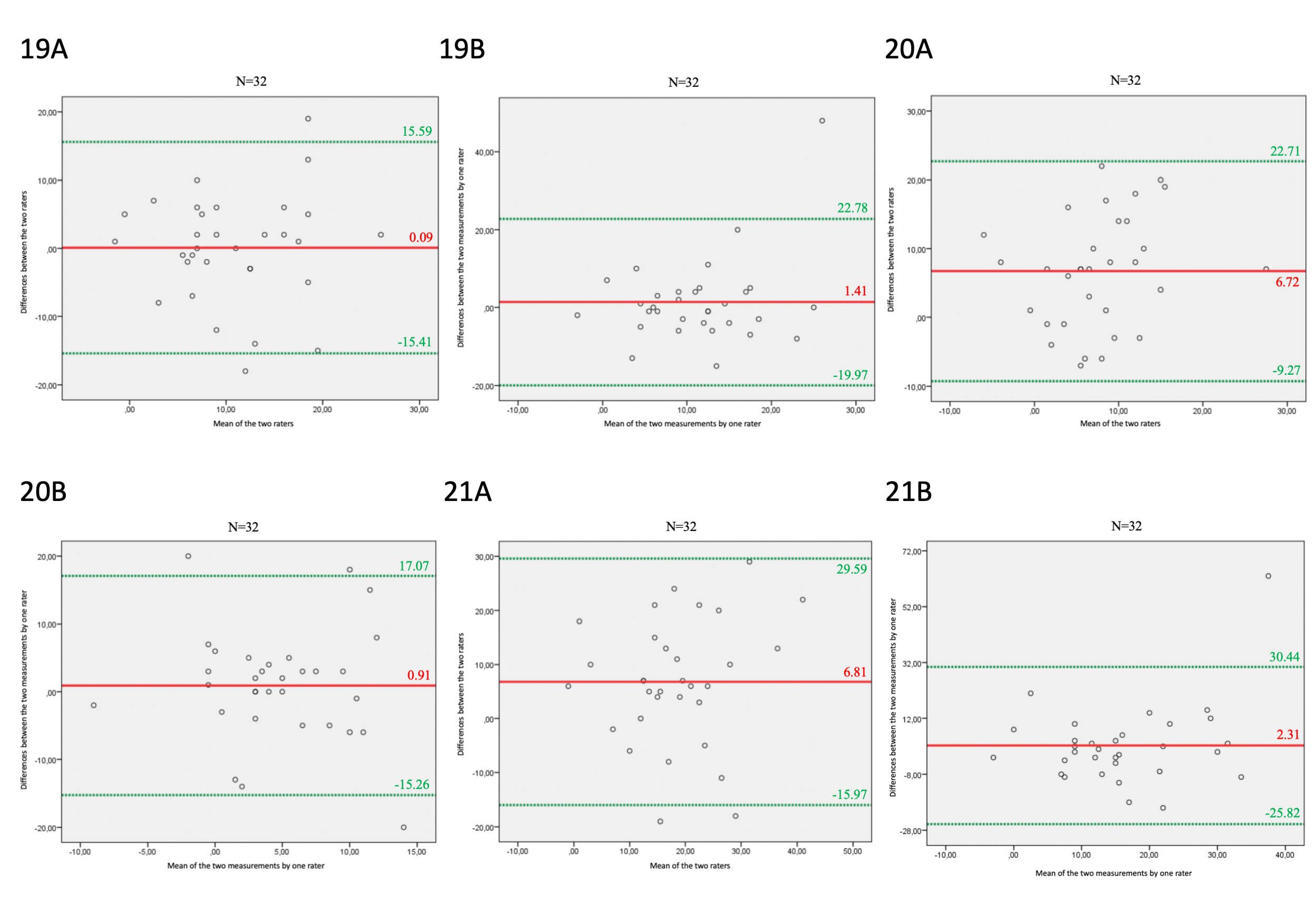

Supplement: Supplementary file 1 — Additional file 1. Additional Figure. 1–7. Bland–Altman plot for agreement of posture inspection in standing (1A and 1B), posture inspection in sitting (2A and 2B), segmental mobility into flexion (3A and 3B), segmental mobility into extension (4A and 4B), posterior to anterior pressure (5A and 5B), inclination of T1–6 in standing (6A and 6B), inclination of T6–12 in standing (7A and 7B), inclination of T1–12 in standing (8A and 8B), inclination of T1–6 in sitting (9A and 9B), inclination of T6–12 in sitting (10A and 10B), inclination of T1–12 in sitting (11A and 11B), C7–T5 flexion mobility (12A and 12B), Schober in neutral (13A and 13B), Schober in flexion (14A and 14B), Schober in extension (15A and 15B), flexion mobility of the T1–6 in sitting (16A and 16B), flexion mobility of the T6–12 in sitting (17A and 17B), flexion mobility of the T1–12 in sitting (18A and 18B), extension mobility of the T1–6 in sitting (19A and 19B), extension mobility of the T6–12 in sitting (20A and 20B) and extension mobility of the T1–12 in sitting (21A and 21B) between raters (A) and within rater (B). The red lines depict the mean difference between raters and dotted green lines depict 95% limits of agreement in the Bland–Altman plot. [file 12891_2020_3551_MOESM1_ESM.zip › Additional figure 7R5.png]
